# Protein–Calixarene Complexation: From Recognition to Assembly

**DOI:** 10.1021/acs.accounts.2c00206

**Published:** 2022-06-06

**Authors:** Peter B. Crowley

**Affiliations:** School of Biological and Chemical Sciences, University of Galway, University Road, Galway H91 TK33, Ireland

## Abstract

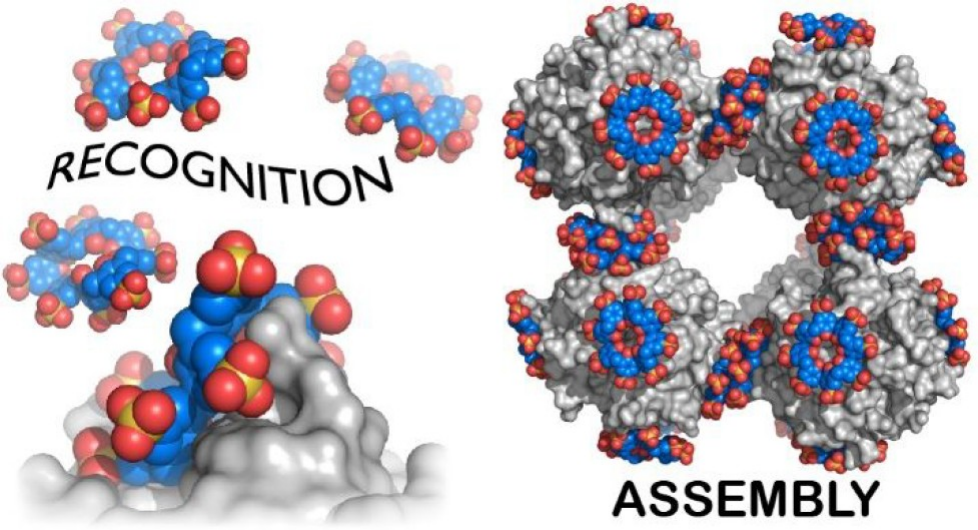

This Account summarizes the progress in protein–calixarene complexation, tracing the developments from binary recognition to the *glue* activity of calixarenes and beyond to macrocycle-mediated frameworks. During the past 10 years, we have been tackling the question of protein–calixarene complexation in several ways, mainly by cocrystallization and X-ray structure determination as well as by solution state methods, NMR spectroscopy, isothermal titration calorimetry (ITC), and light scattering. Much of this work benefitted from collaboration, highlighted here. Our first breakthrough was the cocrystallization of cationic cytochrome *c* with sulfonato-calix[4]arene leading to a crystal structure defining three binding sites. Together with NMR studies, a dynamic complexation was deduced in which the calixarene explores the protein surface. Other cationic proteins were similarly amenable to cocrystallization with sulfonato-calix[4]arene, confirming calixarene–arginine/lysine encapsulation and consequent protein assembly. Calixarenes bearing anionic substituents such as sulfonate or phosphonate, but not carboxylate, have proven useful.

Studies with larger calix[*n*]arenes (*n* = 6, 8) demonstrated the *bigger better binder* phenomenon with increased affinities and more interesting assemblies, including solution-state oligomerization and porous frameworks. While the calix[4]arene cavity accommodates a single cationic side chain, the larger macrocycles adopt different conformations, molding to the protein surface and accommodating several residues (hydrophobic, polar, and/or charged) in small cavities. In addition to accommodating protein features, the calixarene can bind exogenous components such as polyethylene glycol (PEG), metal ions, buffer, and additives. Ternary cocrystallization of cytochrome *c*, sulfonato-calix[8]arene, and spermine resulted in altered framework fabrication due to calixarene encapsulation of the tetraamine. Besides host–guest chemistry with exogenous components, the calixarene can also self-assemble, with numerous instances of macrocycle dimers.

Calixarene complexation enables protein encapsulation, not merely side chain encapsulation. Cocrystal structures of sulfonato-calix[8]arene with cytochrome *c* or *Ralstonia solanacearum* lectin (RSL) provide evidence of encapsulation, with multiple calixarenes masking the same protein. NMR studies of cytochrome *c* and sulfonato-calix[8]arene are also consistent with multisite binding. In the case of RSL, a *C*_3_ symmetric trimer, up to six calixarenes bind the protein yielding a cubic framework mediated by calixarene dimers. Biomolecular calixarene complexation has evolved from molecular recognition to framework construction. This latter development contributes to the challenge in design and preparation of porous molecular materials. Cytochrome *c* and sulfonato-calix[8]arene form frameworks with >60% solvent in which the degree of porosity depends on the protein:calixarene ratio and the crystallization conditions. Recent developments with RSL led to three frameworks with varying porosity depending on the crystallization conditions, particularly the pH. NMR studies indicate a pH-triggered assembly in which two acidic residues appear to play key roles. The field of supramolecular protein chemistry is growing, and this Account aims to encourage new developments at the interface between biomolecular and synthetic/supramolecular chemistry.

## Key References

- RennieM. L.; FoxG. C.; PérezJ.; CrowleyP. B.Auto-regulated Protein Assembly on a Supramolecular Scaffold. Angew. Chem., Int. Ed.2018, 57, 13764–1376910.1002/anie.20180749030109907.^1^*Switch on/switch off oligomerization as a function of protein–calixarene ratio. The first cocrystal structures of a protein and sulfonato-calix[8]arene. Three types of porous frameworks occur, one of which is assembled exclusively by calixarene-mediated interfaces*.
- AlexJ. M.; RennieM. L.; EngilbergeS.; LehoczkiG.; DorottyaH.; FizilÁ.; BattaG.; CrowleyP. B.Calixarene-mediated Assembly of a Small Antifungal Protein. IUCrJ2019, 6, 238–24710.1107/S2052252519000411PMC640018130867921.^2^*Complexation of a ∼ 6 kDa cationic protein with the sulfonato-calix[n]arene (n = 4,6,8) series studied by solution and solid state methods. All three calixarenes bind the same solvent exposed site comprising lysine and hydrophobic side chains. Evidence of dimerization only with n = 8*.
- EngilbergeS.; RennieM. L.; DumontE.; CrowleyP. B.Tuning Protein Frameworks via Auxiliary Supramolecular Interactions. ACS Nano2019, 13, 10343–103503149005810.1021/acsnano.9b04115.^3^*Ternary mixtures of protein, sulfonato-calix[8]arene and spermine result in framework duplication. The tetracationic additive is encapsulated by the calixarene and enables new calixarene-mediated junctions*.
- RambergK. O.; EngilbergeS.; SkorekT.; CrowleyP. B.Facile Fabrication of Protein-Macrocycle Frameworks. J. Am. Chem. Soc.2021, 143, 1896–19073347080810.1021/jacs.0c10697PMC8154523.^4^*The first cocrystal structures of a calixarene and a neutral protein. Two types of porous frameworks are mediated exclusively by sulfonato-calix[8]arene in a pH triggered process*.

## 1 Background

**Figure 1:**
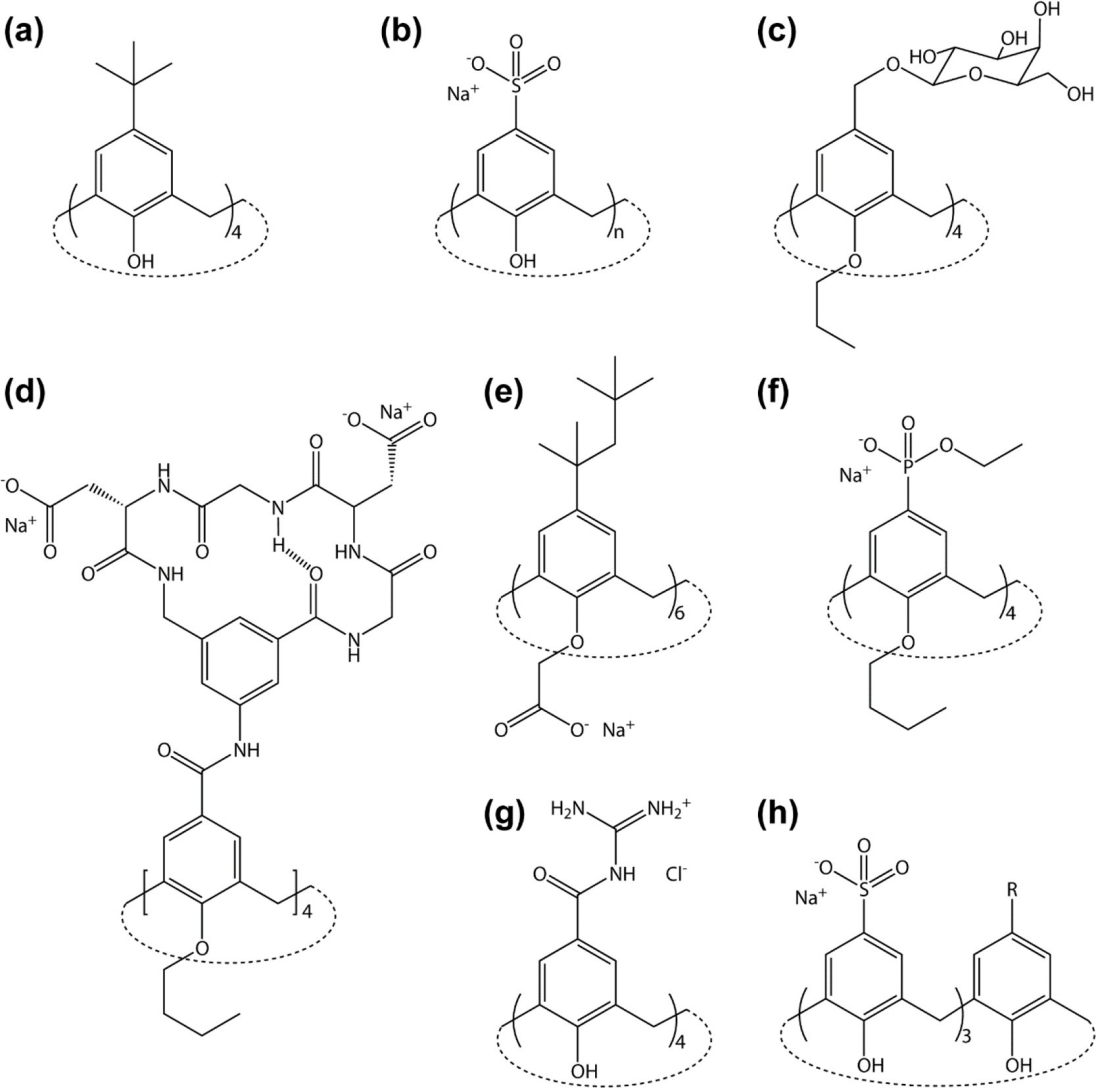
Schematic structures of (a) Gutsche’s canonical calix[4]arene with *t*-butyl groups on the upper rim^5^ and (b–h) water-soluble, protein-binding calixarenes. (b) Shinkai’s sulfonato-calix[*n*]arenes.^6−8^ (c) Representative glyco-calixarene, the tetra-galactoside from Parma.^9^ (d) Hamilton’s peptido-calixarene, containing glycine and aspartate.^10^ (e) Goto’s amphipathic calix[6]arene with lower rim carboxylates.^20^ (f) Schrader’s phosphonate-containing calix[4]arene.^21^ (g) de Mendoza’s guanidinio-calix[4]arene.^24^ (h) Hof’s asymmetric trisulfonato-calix[4]arene.^33^

**Figure 2:**
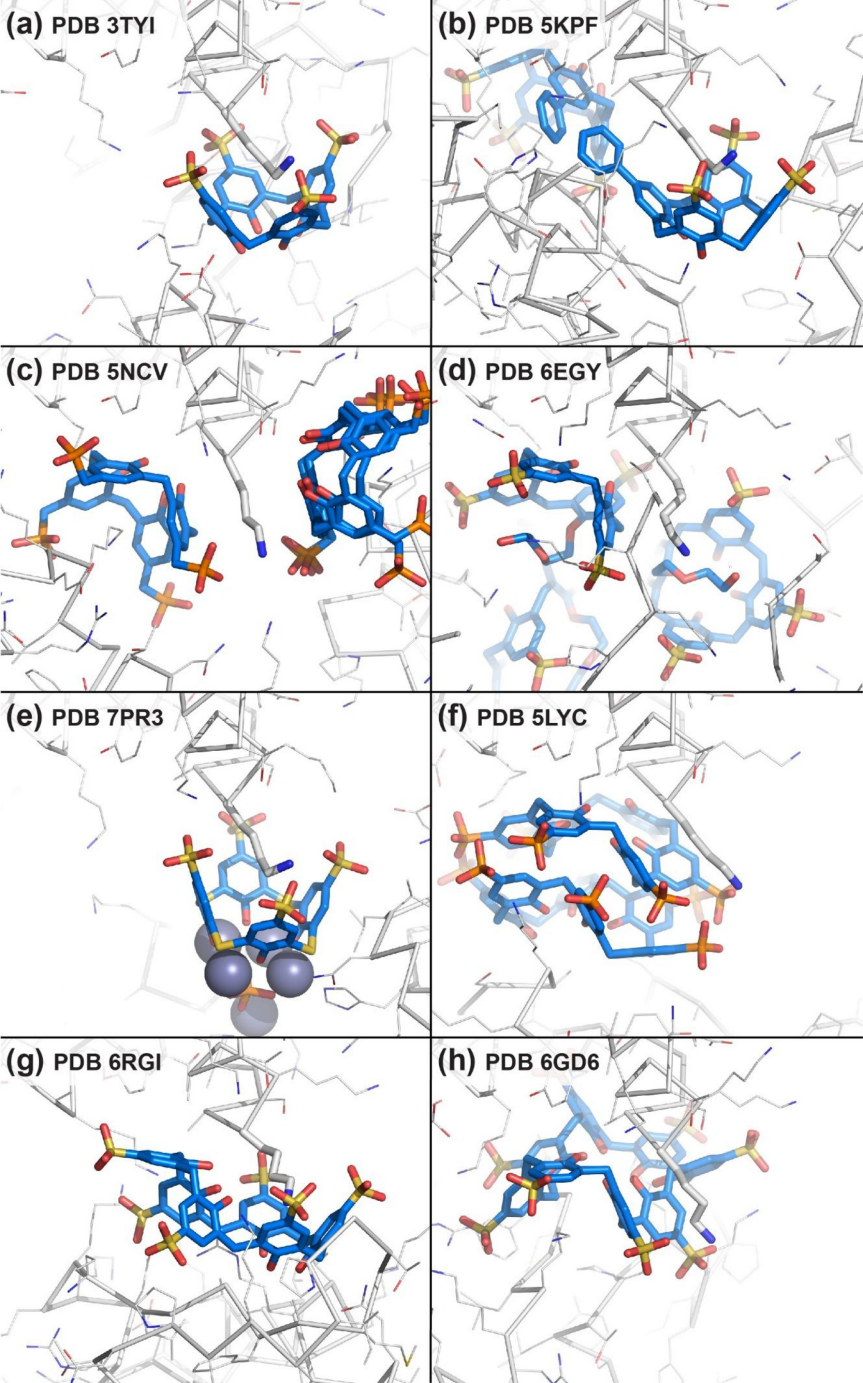
Binding site details from cocrystal structures of cytochrome *c* with (a) sulfonato-calix[4]arene, (b) phenyl-sulfonato-calix[4]arene, (c) methylphosphonato-calix[4]arene, (d) mono-PEGylated sulfonato-calix[4]arene, (e) sulfonato-thiacalix[4]arene with zinc, (f) phosphonato-calix[6]arene, (g) sulfonato-calix[6]arene, and (h) sulfonato-calix[8]arene. Lys4, shown as sticks, is encapsulated or bound *exo* depending on the calixarene.

Calix[*n*]arenes (Figures 1 and 2), cyclic host molecules available in an array of sizes with variable conformations and cavity volumes, were investigated originally as synthetic enzyme mimics.^5^ Molecular recognition, required for substrate binding and possible catalysis, was central to this research. In 1984, Shinkai and co-workers produced a sulfonic acid derivative (Figure 1b) yielding a highly water-soluble calixarene, and demonstrated presently that calixarene cavities were capable of binding guests in water.^6−8^ The mid-1990s onward saw the development of bioinspired calixarenes bearing glyco or peptido features (Figure 1c,d). Here, calix[4]arene served as a rigid scaffold supporting biologic units.^9−12^ These sophisticated receptors were designed for biomolecule recognition, including protein complexation. Hamilton and co-workers reported the first example in which a calixarene bearing four peptide loops bound selectively the lysine-rich cytochrome *c*.^10^ Subsequently, Aoyama and co-workers described calixarene-based saccharide clusters that agglutinated lectins.^11^

Fifteen years after Shinkai’s seminal work, there appeared the first reports on sulfonato-calix[4]arene complexation of amino acids including arginine and lysine or short peptides thereof.^13−18^ These studies affirmed the potential of simple calixarenes as protein receptors. Small molecule X-ray crystal structures were particularly instructive, revealing partial encapsulation of the amino acid side chain in the calix[4]arene cavity.^14,16,17^ During the 2000s, multisite binding between sulfonato-calix[*n*]arenes and bovine serum albumin (BSA) was reported, leading to protein precipitation in salt-free solutions.^19^ Multisite calixarene–protein binding was suggested also by elegant experiments from Goto and co-workers who transferred cytochrome *c* from water into chloroform by complexation with an amphipathic calix[6]arene (Figure 1e).^20^ Schrader and co-workers used amphipathic calix[4]arenes for protein sensing.^21,22^ Lower rim butoxy groups enabled membrane-embedment while the upper rims were functionalized with amino or phosphonate groups (Figure 1f) to complement the charged properties of the target protein. In 2008, protein–calixarene complexation was given a twist. Rather than avail of the macrocycle cavity for side chain encapsulation, de Mendoza and co-workers plugged the cavities of the p53 tetramerization domain with a calix[4]arene.^23^ Guanidinium functionalities on the calixarene rescued an arginine to histidine mutation and stabilized the protein. The plug concept was taken further with complete matching between the *C*_4_ symmetric potassium channel and guanidinio-calix[4]arenes (e.g., Figure 1g).^24^ Meanwhile there were further developments with multivalent glyco-calixarenes^25^ and calixarene-based protein inhibitors.^26−29^

In 2010, Hof and co-workers revisited sulfonato-calix[4]arene complexation of the cationic amino acids.^14,15,17,30,31^ The focus was on arginine/lysine methylation. Modification of the lysine-^ζ^NH_3_^+^ primary amine to the mono-, di-, and trimethylated forms increased the binding affinity for sulfonato-calix[4]arene (e.g., ∼70-fold tighter for trimethyllysine).^27^ A tetrapeptide snippet from the disordered N-terminus of histone H3 had ∼18-fold increased affinity for sulfonato-calix[4]arene when the lysine side chain was trimethylated. In a subsequent study, longer H3 peptides had micromolar affinities for the calixarene, acting akin to the aromatic cage motif of histone reader proteins.^32^ Improved affinity and selectivity toward trimethyllysine were obtained using trisulfonated calix[4]arenes (Figure 1h).^33^

With this overview of the key stepping stones, we turn now to our investigation of calixarene complexation, in particular X-ray cocrystal structures with model proteins (Tables 1 and 2). Numerous reviews are available for further insights to the past developments and the current biological applications of calixarenes.^34−36^

**Table 1:** Model Protein Characteristics

protein, organism	oligomer	fold	MW (kDa)	#Lys	#Arg	p*I*_calc_
cytochrome *c*, *S. cerevisiae*	monomer	all alpha heme core	12.8	16	3	9.5
Lysozyme, *G. gallus*	monomer	alpha and beta two domain	14.3	6	11	9.3
PAF, *P. chrysogenum*	monomer	small protein disulfide-rich	6.2	13	0	8.9
PAFB, *P. chrysogenum*	monomer	small protein disulfide-rich	6.5	8	2	8.8
RSL, *R. solanacearum*	trimer	beta-propeller 6-blades	29.1	9	9	6.8

**Table 2:** Protein–Calixarene Crystal Structures in the Protein Data Bank

PDB ID	space group	protein	ligand
3TYI	*P*2_1_2_1_2_1_	cyt *c*	sulfonato-calix[4]arene
5LFT	*P*22_1_2_1_	cyt *c*	bromo-sulfonato-calix[4]arene
5KPF	*C*222_1_	cyt *c*	phenyl-sulfonato-calix[4]arene
5NCV	*P*12_1_1	cyt *c*	methylphosphonato-calix[4]arene
6EGY	*I*4_1_32	cyt *c*	sulfonato-calix[4]arene monoPEG
6EGZ	*I*4_1_32	cyt *c*	sulfonato-calix[4]arene diPEG
6SUV	*P*4_3_	cyt *c*^a^	octa-anionic-calix[4]arene
6SUY	*P*3_2_21	cyt *c*	octa-anionic-calix[4]arene
7PR3	*P*2_1_2_1_2_1_	cyt *c*	sulfonato-thiacalix[4]arene + Zn
5LYC	*P*4_3_2_1_2	cyt *c*	phosphonato-calix[6]arene
6RGI	*P*3_2_21	cyt *c*	sulfonato-calix[6]arene
6GD6	*H*3	cyt *c*	sulfonato-calix[8]arene
6GD8	*P*3_1_	cyt *c*	sulfonato-calix[8]arene
6GD9	*P*4_3_2_1_2	cyt *c*	sulfonato-calix[8]arene
6RSK	*P*4_3_2_1_2	cyt *c*	sulfonato-calix[8]arene + spermine
6Y0J	*P*6_1_	cyt *c*	calix[6]arene and calix[8]arene
7BBT	*C*121	cyt *c*	extended arm calix[8]arene
4PRQ	*P*12_1_1	lysozyme	sulfonato-calix[4]arene
4N0J	*P*12_1_1	lysozyme^b^	sulfonato-calix[4]arene
6HA4	*P*12_1_1	PAF	sulfonato-calix[4]arene
6HAH	*P*12_1_1	PAF	sulfonato-calix[6]arene
6HAJ	*P*6_1_	PAF	sulfonato-calix[8]arene
7BAD	*P*3_1_	PAFB	sulfonato-calix[8]arene
7PR5	*P*2_1_2_1_2_1_	RSL	sulfonato-thiacalix[4]arene + Zn
6Z5X	*P*2_1_3	RSL	sulfonato-calix[8]arene
6Z5G	*I*23	RSL	sulfonato-calix[8]arene
6Z5Q	*P*3	RSL	sulfonato-calix[8]arene
6Z5P	*P*3	RSL-R_8_	sulfonato-calix[8]arene

a *Equus caballus* cytochrome *c*.

b Dimethylated protein with R–NH_3_^+^ converted to R–NH(CH_3_)_2_^+^.

## 2 First Steps with Sulfonato-calix[4]arene

### 2.1 The Complex of Cytochrome *c* and Sulfonato-calix[4]arene

One strategy for protein surface recognition involves synthetic receptor molecules with a hydrophobic core enabling a water-occluded interface, and a polar/charged periphery complementing the charged features of the protein.^10,12,21,37−39^ This concept was illustrated beautifully by Aya and Hamilton (Figure 1 in ref (37)) who reported anionic porphyrins with nanomolar affinity for cytochrome *c*. Other proteins with different surface attributes could be targeted using porphyrins bearing the appropriately charged functionality.^37,38^ Following this line of research we obtained NMR data suggesting nonspecific binding between two anionic porphyrins and *Saccharomyces cerevisiae* cytochrome *c*.^39^ Attempts to cocrystallize these complexes were fruitless. Replacing the planar porphyrin with the bowl-shaped calix[4]arene proved to be a game-changer.

In Autumn 2010, we began cocrystallization trials of cytochrome *c* and sulfonato-calix[4]arene (745 Da), the latter provided by colleague Nicholas Power. Our idea was to maximize the protein–calixarene attraction by maintaining a low ionic strength. Therefore, PEG 8000 was used as a precipitant in the absence of buffer or salt. That year, the International Conference on Crystallization of Biological Macromolecules (ICCBM13) was held in Dublin. On returning from the conference, PhD student Róise McGovern emerged excitedly from the laboratory. Her first trial had yielded crystals! The X-ray diffraction images, obtained in collaboration with Amir Khan, contained smeared and overlapping spots, *ugly but promising*. Optimization involved adjusting the salt composition and preparing homogeneous protein–calixarene mixtures. Eventually, high-quality diffraction data were obtained at the European Synchrotron Radiation Facility (Grenoble). Meanwhile, we had collected multiple NMR data sets. HSQC-monitored titrations of ^15^N-labeled protein indicated a lysine-rich binding patch that accommodated at least two calixarenes with millimolar affinities.

The crystal structure of the cytochrome *c*–sulfonato-calix[4]arene complex was informative for three reasons.^40^ (1) The structure proved unambiguously that calix[4]arene was capable of protein complexation by encapsulation (*endo* binding) of one lysine side chain (Figure 2a). Of the three crystallographic sites, Lys4 and Lys89 were consistent with the NMR data while Lys22 was not, suggesting that it arose via crystal packing. (2) The occurrence of three binding sites confirmed the concept of multisite protein–calixarene complexation.^19,20^ We suggested that the calixarene could hop between lysine side chains and *camouflage* the protein surface. (3) In the crystal, each calixarene occurred as a junction between two or more proteins. *Exo* interactions with lysines and other side chains resulted in this *glue* activity, altering the protein solubility in favor of assembly/crystallization. Apparently, calix[4]arene complexation of lysine, with interfaces of ∼200 Å^2^, is an example of surface-entropy reduction facilitating protein crystallization.^41^ In addition to clamping down a lysine, the calixarene converts a heterogeneous protein surface to a *C*_4_ symmetric cap. Prior to publication, I presented this work at the 2011 Bürgenstock Conference, where Ivan Huc and Tom Fyles offered great encouragement.

Unknown to us at the time, Falson, Coleman, and co-workers had earlier reported an asymmetric carboxylato-calixarene bearing a lipid for the extraction and purification of membrane proteins.^42^ This surfactant calixarene was cocrystallized with a *Bacillus* ABC transporter, but the diffraction data were insufficient to detect the macrocycle. Subsequently, a calixarene-containing crystallization kit was commercialized by CALIXAR (section 3.2).

Cytochrome *c*–sulfonato-calix[4]arene crystals are robust, an attribute which makes them attractive for applications. Through an EU Cost Action, I met Fred Lisdat who was constructing multilayer electrodes of cytochrome *c* and polyaniline sulfonate.^43^ The possibility of replacing polyaniline sulfonate with sulfonato-calix[4]arene was immanent. In collaboration, we grew cocrystals of cytochrome *c* and sulfonato-calix[4]arene on modified gold chip electrodes and obtained direct electrochemical characterization by cyclic voltammetry.^44^

### 2.2 Cocrystals of Lysozyme and Sulfonato-calix[4]arene

**Figure 3:**
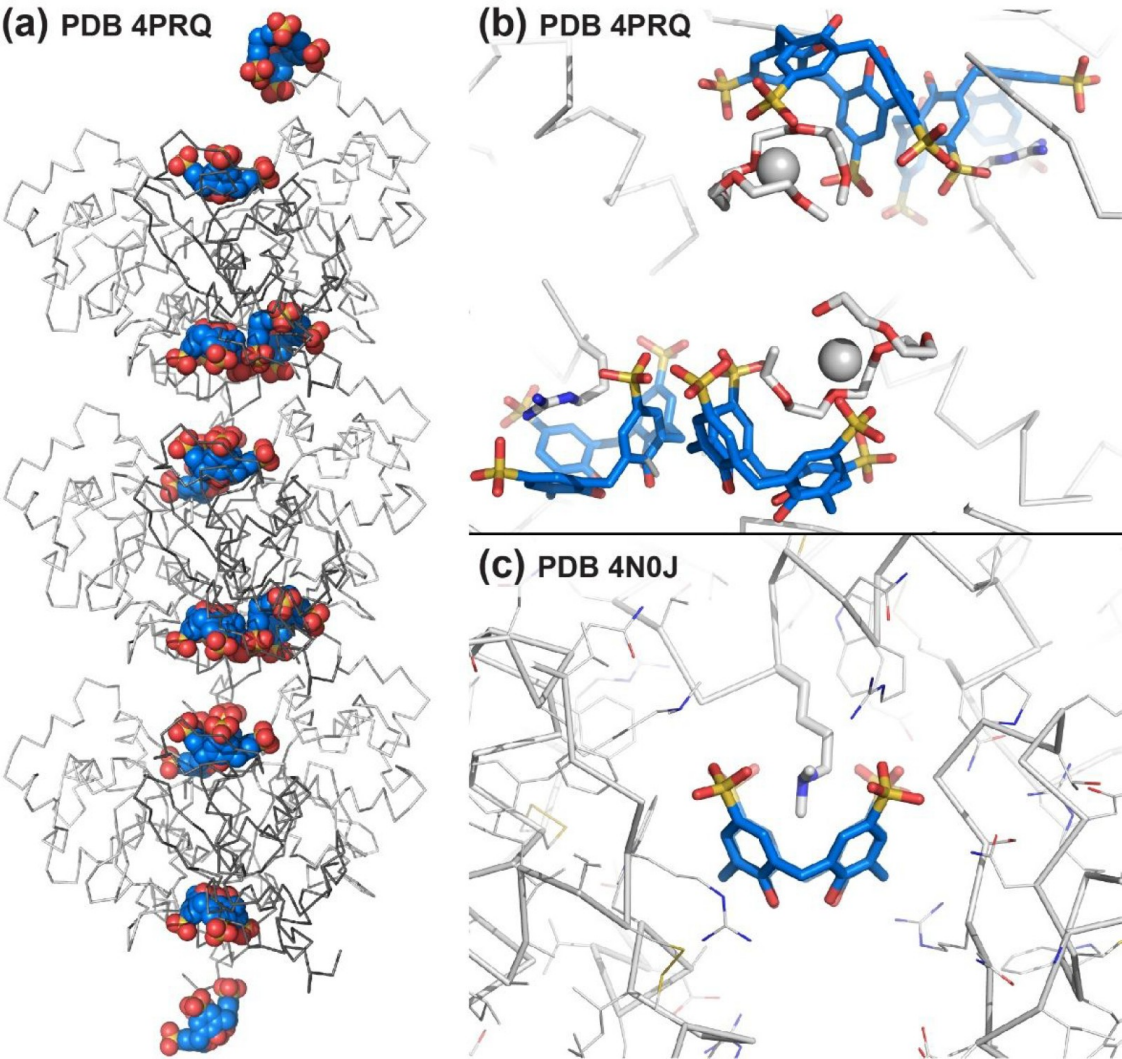
(a) Filament of lysozyme tetramers (three shown) mediated by dimers of sulfonato-calix[4]arene and (b) detail of the calixarene dimer with encapsulation of Arg128 and a complex of Mg^2+^ and PEG. (c) Cocrystal structure of dimethylated lysozyme and sulfonato-calix[4]arene showing encapsulation of Lys116*.

The easily crystallizable hen egg white lysozyme was an obvious target for sulfonato-calix[*n*]arene complexation. Similar to cytochrome *c*, lysozyme has an isoelectric point (p*I*) of ∼9 but it is arginine-rich rather than lysine-rich (Table 1). The combination of lysozyme and sulfonato-calix[4]arene in water resulted in instantaneous precipitation.^45^ Similar crystallization conditions to those used for cytochrome *c*(40) formed heavy precipitates that eventually yielded small cubic crystals. X-ray diffraction was performed at SOLEIL synchrotron (Gif-sur-Yvette, France) in collaboration with Andrew McCarthy. The lysozyme–sulfonato-calix[4]arene cocrystal structure comprised a *D*_2_-symmetric tetramer arranged in filaments (Figure 3).^45^ The lysozyme tetramer had a central channel (∼10 Å diameter) plugged at either end by a pair of calixarenes with their cavities projected outward, reminiscent of earlier work on calix[4]arenes with tetrameric channels.^23,24^ Interestingly, N-terminal Lys1 of lysozyme was bound *exo* to the calixarene dimer. One calixarene encapsulated the side chain of Arg128 from a neighboring molecule. Located in the C-terminus, Arg128 is the most sterically accessible of the 11 arginines in lysozyme. The other calixarene complexed a magnesium ion and a fragment of PEG, behaving like a crown ether (Figure 3b). This entity was supported by data from Raston and co-workers who had described structures of sulfonato-calix[6]arene, 18-crown-6 and lanthanides.^46^

The same year that we reported calix[4]arene-mediated assembly of lysozyme, Yang and co-workers reported that sulfonato-calix[4]arene inhibits pentamer formation by capsid protein L1 (p*I* ∼ 9) from human papillomavirus.^47^ In 2016, supramolecular assembly of sulfonato-calix[4]arene and cationic protamine was described by Liu and co-workers,^48^ while Mohanty and co-workers used sulfonato-calix[4]arene to inhibit insulin amyloidogenesis.^49^

### 2.3 Lysine Methylation and Sulfonato-calix[4]arene

Considering the enhanced affinity for dimethyllysine over lysine,^31^ we cocrystallized sulfonato-calix[4]arene with dimethylated lysozyme (lysozyme*). The cocrystal structure revealed calixarene complexation of Lys116*, the most sterically accessible such group (Figure 3c).^50^ The binding mode, with a pronounced cation-pi contribution, contrasted to that of unmodified lysine. The selectivity for Lys116* was supported by an NMR study performed by Fraser Hof and co-workers. In addition to Lys116* complexation, the crystal structure revealed binding to Arg14. This result emphasized that crystal packing can produce binding sites that do not occur in solution. For example, two lysozyme molecules interacted *exo* to the calixarene at Arg14, with salt bridges formed between the lower rim phenols and Arg21.

Meanwhile, calixarene-functionalized agarose resin was developed for peptide purification based on lysine methylation.^51^ Zhong and co-workers devised a related strategy by host-assisted capillary electrophoresis.^52^ In addition to sulfonato-calix[*n*]arenes, related macrocycles were developed as Lys(Me)_*n*_ receptors.^53,54^

## 3 Cytochrome *c* Complexation with Other Calix[4]arenes

### 3.1 Asymmetric Trisulfonato-calixarenes

Working with trisulfonato-calixarenes from the Hof laboratory,^33^ MSc student Aishling Doohan tested if host asymmetry altered the binding specificity. The bromo derivative (Figure 1h, R = Br, 744 Da) cocrystallized readily with cytochrome *c* under conditions similar to those reported previously.^40,55^ The crystal structure yielded evidence for selective binding, as only Lys86 was encapsulated. The bromo substituent made van der Waals contact with the Lys86 backbone carbonyl, raising the possibility of halogen bonding. On the other hand, there was evidence also of nonspecific binding.^55^ A calixarene dimer,^56^ with encapsulated bromo substituents, occurred at 70% occupancy wedged between two protein chains. One protein used Lys5 and Lys89, while the other protein used Lys4 and Lys100 to bind this calixarene dimer via cation−π bonds.

Cocrystals of the phenyl derivative (Figure 1h, R = C_6_H_5_, 741 Da) and cytochrome *c* were obtained by microseeding with cytochrome *c*–sulfonato-calix[4]arene cocrystal seeds.^55^ The crystal structure revealed a single site with Lys4 bound *endo* to the calixarene. Interestingly, the phenyl substituent made van der Waals contact with Ala3 and a calixarene bound to a symmetry mate in the crystal packing (Figure 2b). This weak calixarene dimerization at a protein–protein interface suggested a mechanism for protein–calixarene aggregation observed in buffered solutions. While the X-ray data revealed variations in specificity of the trisulfonato-calix[4]arenes, the NMR data suggested a broad binding patch, though not as extensive as for sulfonato-calix[4]arene. Thermodynamic analysis, by ITC, yielded two site binding and apparent dissociation constants of 0.02 and 0.03 mM for the bromo derivative and for sulfonato-calix[4]arene, respectively. The phenyl derivative resisted ITC analysis due to aggregation.

### 3.2 Anionic Calix[4]arenes from Parma

PhD student Jimi Alex tested the CALIXAR kit^42^ containing calix[4]arenes variously functionalized with carboxylato or phosphonato substituents at the upper or lower rims. No cocrystals were obtained with the carboxylato derivatives, a result borne out by other carboxylato-macrocycles^57,58^ that have resisted cocrystallization. Only the upper rim methylphosphonato-calix[4]arene yielded cocrystals with cytochrome *c*. This compound, synthesized originally by Ungaro and co-workers added impetus to our ongoing collaboration with Alessandro Casnati.^81^ Cocrystallization occurred at 2 equiv of this ligand, compared to the 10 equiv required for sulfonato-calix[4]arene. A crystal structure of methylphosphonato-calix[4]arene (800 Da) in complex with cytochrome *c* was instructive for several reasons.^59^ Lys86 was selected as the binding site, similar to the complex with the bromo derivative. One of the methylphosphonato substituents rotated into the cavity affording new interactions between the encapsulated cation and the upper rim anion. A second binding site at Lys54, was likely a result of crystal packing as it was not evident in NMR experiments. Within the crystal packing, key residue Lys4, was sandwiched *exo* between two calixarenes (Figure 2c).

Jimi Alex investigated another calix[4]arene from Parma, with upper rim sulfonato- and lower rim carboxylato- groups. This octa-anionic calixarene (977 Da) is locked in the cone conformation by lower rim coordination of a Na^+^ ion. Silvano Geremia and co-workers obtained cocrystals with horse cytochrome *c* making for a comparison with our data on the yeast protein.^60^ In both cases the calixarene bound to charge rich patches and yielded porous assemblies (section 7). Curiously, in the yeast case, the calixarene did not encapsulate any side chain.

### 3.3 PEGylated Calix[4]arenes

Postdoc Srinu synthesized sulfonato-calix[4]arenes bearing one, two, or four PEG chains. Our goal was supramolecular PEGylation of proteins, using the calixarene to tether the PEG to the protein. As noted in 2016, a related concept was developed originally with PEGylated triazine dyes to improve enzyme solubility.^61^ While our work was in progress, Isaacs, Langer, Anderson, and co-workers reported a system based on PEGylated cucurbit[7]uril.^62^ Working with cytochrome *c* and the mono- or di-PEGylated calix[4]arene (1.5 and 1.9 kDa, respectively), Srinu obtained crystallographic proof of supramolecular PEGylation.^63^ The protein–calixarene binding sites were familiar but the PEG appendages gave rise to new features. In the mono-PEG case, the calixarene occurred in either the cone or partial cone conformation (Figure 2d). Self-encapsulation of a PEG fragment together with Mg^2+^ occurred also, similar to earlier observations.^45^

### 3.4 Thiacalix[4]arene

Recently, we demonstrated the capacity of sulfonato-thiacalix[4]arene (816 Da) for macrocycle- and metal-mediated protein assembly.^64^ PhD student Ronan Flood obtained cocrystals of two model proteins and sulfonato-thiacalix[4]arene in combination with zinc. In cocrystals with cytochrome *c*, the thiacalixarene supported penta-nuclear zinc clusters that acted as nodes for protein complexation (Figure 2e). Due to space limitations, we direct the reader to the paper for full details.^64^

## 4 Bigger Better Binders

### 4.1 Protein Dimerization with Phosphonato-calix[6]arene

Shortly after the 12th International Conference on Calixarenes, I received a package of phosphonato-calix[*n*]arenes from Colin Raston.^65^ A cocrystal structure of phosphonato-calix[6]arene (1117 Da) and cytochrome *c*, together with solution state studies by postdoc Martin Rennie, marked another turning point in our research.^66^ The crystal structure contained a calix[6]arene dimer. Each calixarene bound one protein by encapsulating Lys4, Lys100, and a small hydrophobic patch to form an ∼350 Å^2^ interface (Figure 2f). A porous assembly occurred with an ∼60% solvent content (compared to the 30–40% in typical protein crystals). The interface area of protein–protein contacts was approximately equal to that of protein–calixarene and calixarene–calixarene contacts, suggesting a pivotal role for the calixarene within the assembly. Importantly, the calixarene-mediated protein dimer was not merely a consequence of crystal packing. Three different solution state techniques, NMR spectroscopy, size exclusion chromatography coupled with multiangle light scattering (SECMALS), and ITC, pointed to the existence of dimers and higher order oligomers. Furthermore, the ITC data indicated a binding affinity in the low micromolar range. This improved affinity, with respect to calix[4]arene, may be attributed in part to the larger interface size.^66^

Curiously, sulfonato-calix[6]arene (1117 Da) did not exert the same effects on cytochrome *c*. In this case, crystals were obtained in the presence of imidazole, which displaced the Met80 heme ligand resulting in a partially unfolded cytochrome supported by calix[6]arene complexation (Figure 2g).^67^

### 4.2 Protein Assembly and Encapsulation with Sulfonato-calix[8]arene

**Figure 4:**
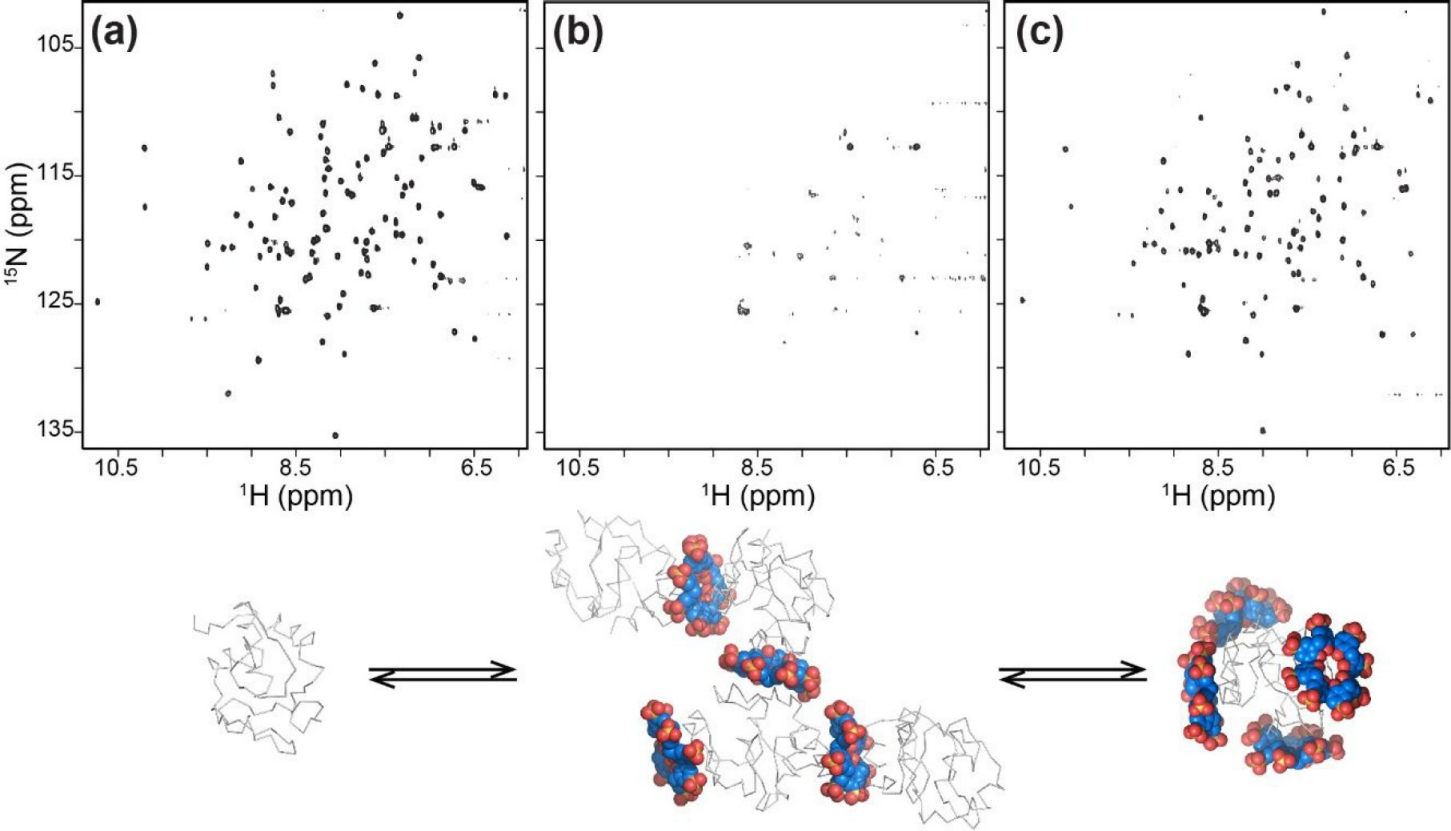
NMR and X-ray data suggest autoregulated assembly of cytochrome *c* by sulfonato-calix[8]arene. ^1^H–^15^N HSQC spectra of (a) pure protein and protein plus (b) ∼1 and (c) ∼3 equiv of calixarene. Spectral obliteration may be due to the formation of high molecular weight species such as a tetramer (based on PDB 6GD8). Higher equivalents of ligand result in masking/encapsulation (based on PDB 6GD9), disassembly, and spectral recovery.

The *bigger better binder* theme comes to the fore with sulfonato-calix[8]arene (1489 Da). Long before a crystal structure was available, we had NMR data pointing to unusual behavior of cytochrome *c* and sulfonato-calix[8]arene (Figure 4). The ^1^H–^15^N HSQC spectrum of cytochrome *c* was obliterated by ∼1 equiv of the macrocycle.^1^ In itself, this result is not unusual as ligand-mediated aggregation may result in NMR-invisible high molecular weight species. However, further additions of macrocycle resulted in spectral improvement, and at ∼3 equiv the spectrum was restored albeit with multiple chemical shift perturbations. The existence of a high molecular weight species, specifically a tetramer, was evident from light scattering measurements including small-angle X-ray scattering (SAXS) data obtained in collaboration with Javier Pérez (SWING beamline, SOLEIL synchrotron). Biphasic ITC data that could not be fitted to a bidentate ligand model were also consistent with a multispecies process.^68^ Therefore, it appeared that the calix[8]arene could switch on and off protein assembly. Such autoregulated assembly is characteristic of cytokine–heparin interactions as well as the assembly of cationic proteins by polyphosphates.^1^

Cocrystallization trials with cytochrome *c* and sulfonato-calix[8]arene initially proved ineffective but revealed the counterintuitive observation that the degree of precipitation was inversely proportional to the calixarene concentration. Martin Rennie made the breakthrough, and *as if seed crystals flew*(69) cocrystallization with sulfonato-calix[8]arene is now straightforward. Three crystal forms of cytochrome *c* in complex with sulfonato-calix[8]arene lent support for a tetramer assembly (Figures 2h and 4) and revealed three essential properties: (1) *promiscuity of binding*, where five distinct calix[8]arene-binding patches were evident, involving interface areas up to ∼500 Å^2^ and varying degrees of macrocycle molding to the surface of cytochrome *c*; (2) *protein masking/encapsulation*, where each structure included two or more sulfonato-calix[8]arene binding sites with up to ∼35% surface coverage suggesting a mechanism for disassembly of high molecular weight species as deduced from the NMR experiments (protein encapsulation, Figure 4), and (3) *porous framework fabrication*, where all three structures were highly porous, with solvent contents > 60%. These frameworks were elaborated by the addition of spermine (section 7).^3^

### 4.3 Alternative Assembly by an “Extended Arm” Calix[8]arene

Aspiring to affect even greater protein surface coverage (encapsulation), we worked with an “extended arm” calix[8]arene from Colin Raston’s laboratory. PhD student Niamh Mockler cocrystallized this 2.2 kDa macrocycle bearing benzyl extensions with cytochrome *c*. Although improved encapsulation beyond the capacity of sulfonato-calix[8]arene was hypothesized, we obtained a novel binding mode by a supramolecular entity.^70^ The crystal included a trimeric stack of the extended arm calixarene. Remarkably, the benzyl extensions projected from this stack forming four grooves, each of which accommodated the N-terminal α helix of cytochrome *c*. Apparently, bigger is not always better. The structure was further surprising in that the calixarene stack was threaded by a PEG fragment yielding a pseudorotaxane.

### 4.4 Mixed Calixarenes for Protein Crystallization

To add a final layer of complexity, consider the composite structure of cytochrome *c* with phosphonato-calix[6]arene and sulfonato-calix[8]arene. Ternary cocrystals, obtained at an approximately 1:1:1 ratio of the components, involved two types of calixarene-mediated interfaces.^71^ Phosphonato-calix[6]arene formed a dimer and bound two molecules of cytochrome *c* via the Lys4 and Lys100 pair. Interestingly, the direction of rotation within this dimeric assembly was opposite to that in the original structure.^66^ Sulfonato-calix[8]arene also bound a known site (Lys72, Lys73, and Lys86)^1^ and again mediated a cytochrome *c* dimer. The crystal packing was a highly porous (70% solvent content), dendrite-like assembly of supramolecular copolymers with alternating phosphonato-calix[6]arene or sulfonato-calix[8]arene junctions as well as one protein–protein interface.^71^

## 5 Antifungal Proteins

**Figure 5:**
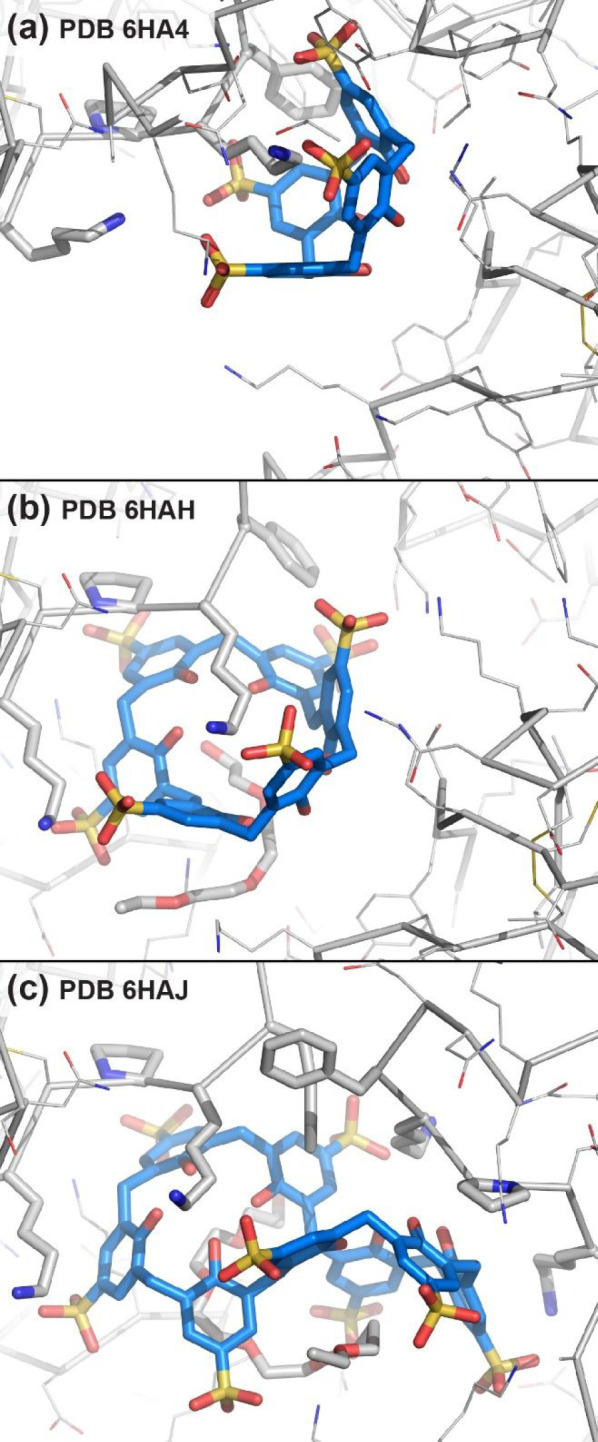
Binding sites in cocrystal structures of PAF with (a) sulfonato-calix[4]arene, (b) sulfonato-calix[6]arene, and (c) sulfonato-calix[8]arene. Lys27, Pro29, Lys30, and Phe31 shown as sticks. Note PEG fragments in (b) and (c).

At the 2016 Chianti Workshop, Gyula Batta presented his NMR studies of *Penicillium* antifungal protein (PAF).^72^ This small and highly cationic protein immediately caught our attention as a candidate for calixarene complexation. Jimi Alex took on the challenge and her work proved seminal (Figure 5). Simple conditions, comprising only PEG and a buffer,^40,59^ resulted in cocrystallization of PAF with the sulfonato-calix[*n*]arene series, while PAF alone did not yield diffraction-quality crystals.^2^ The cocrystal structures of PAF with sulfonato-calix[4]arene or sulfonato-calix[6]arene were solved in the same space group (*P*12_1_1) with similar unit cell parameters and one calixarene per polypeptide. In contrast, the cocrystal with sulfonato-calix[8]arene was hexagonal with one calixarene per two proteins. The same highly exposed feature (Pro29, Lys30, and Phe31) bound the calix[*n*]arene in each structure. Adopting the double cone conformation, sulfonato-calix[8]arene interacted *exo* to the Pro29/Lys30/Phe31 patch on two PAF molecules. In the crystal packing, each calix[*n*]arene bound to at least four proteins, substantiating the *glue* activity (Figure 5). A fragment of PEG completed the protein–calixarene binding sites in the calix[6]arene and calix[8]arene structures. The latter was threaded with a heptaethylene glycol unit forming extensive interactions with the calixarene as well as crown-ether-like complexes with Lys9 residues. The PAF–sulfonato-calix[*n*]arene binding sites identified by X-ray crystallography were confirmed in solution by NMR spectroscopy. Micromolar binding affinities were determined by ITC and the tightest binding sulfonato-calix[8]arene acted as a bidentate ligand, consistent with the X-ray model of ligand-mediated dimerization.^2^

In a follow-up study, we collaborated with Florentine Marx and co-workers to study the histidine-rich PAFB with ∼35% sequence identity to PAF. Postdoc Francesca Guagnini obtained diffraction-quality crystals of PAFB with sulfonato-calix[8]arene in the presence of zinc. The cocrystal structure (space group *P*3_1_) was highly porous and included a trinuclear Zn cluster, assembled by three proteins each contributing two histidines.^73^ The adjacent calix[8]arene binding site, masked ∼400 Å^2^ of the protein surface.

## 6 A Symmetric, *Neutral* Target—Toward Encapsulation

**Figure 6:**
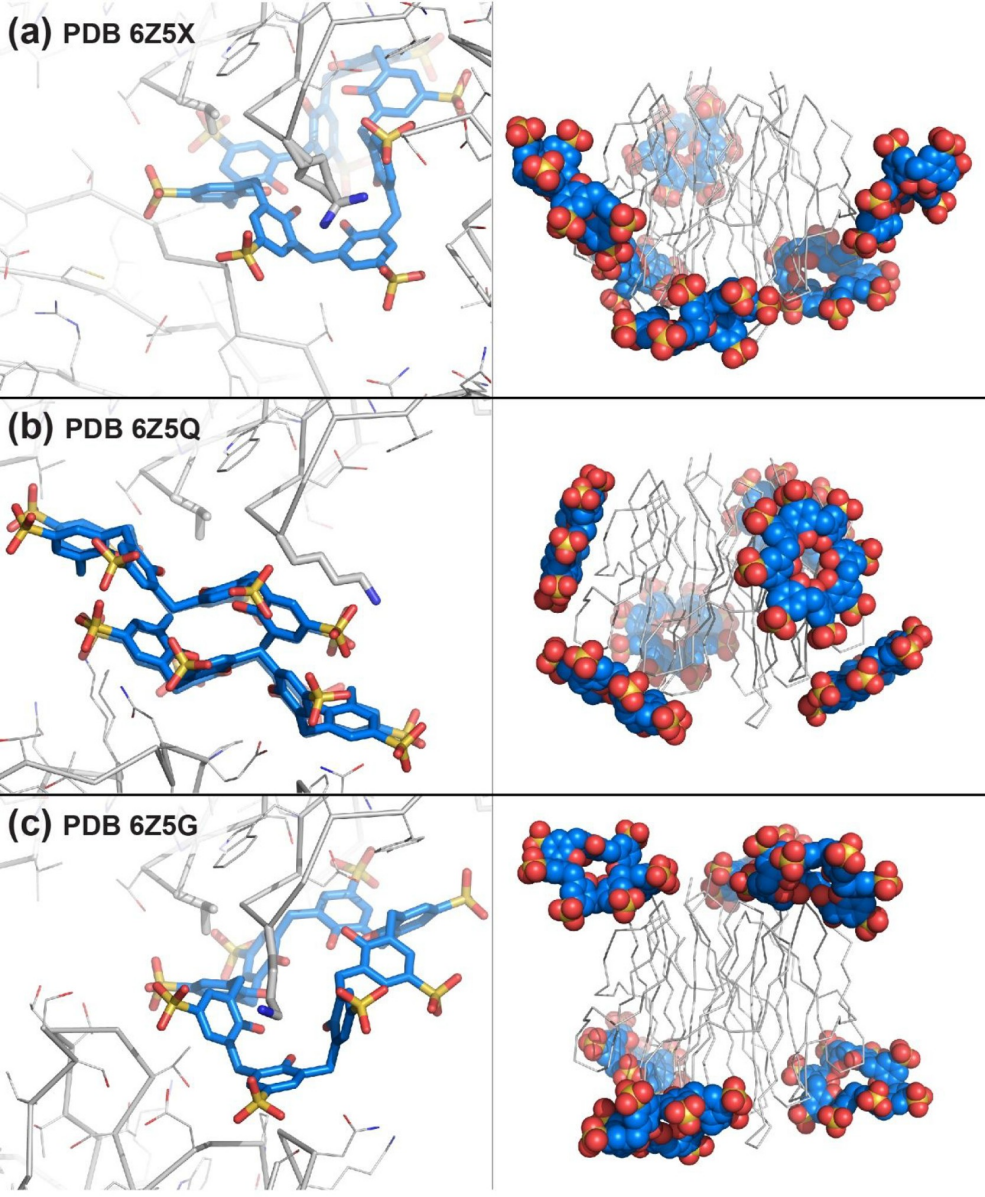
Binding sites and surface masking/encapsulation in three cocrystal structures of RSL with sulfonato-calix[8]arene, space groups (a) *P*2_1_3, (b) *I*23 and (c) *P*3. The recurring epitope, Val13 and Lys34, shown as sticks. Note the disorder Lys34 in (a).

Thus far, we have discussed cationic targets.^1,2,45,50^ Until recently, there was limited evidence for calixarene complexation of acidic proteins.^19,21,49^ NMR studies revealed sulfonato-calix[4]arene complexation of arginine and/or lysine side chains in human ubiquitin^74^ and the WW domain of peptidyl-prolyl isomerase Pin1, each with a p*I* < 7.^75^ What about a *neutral* protein? RSL is a highly stable *C*_3_ trimer with a 6-bladed β-propeller topology and a p*I* close to 7. The absence of a cationic patch and the high symmetry made RSL an interesting target for calixarene complexation. Postdoc Sylvain Engilberge made the first progress obtaining cocrystals at >30 equiv of sulfonato-calix[8]arene in high concentrations of ammonium sulfate across a wide pH range.^4^ These results suggested that charge–charge interactions were minimal, and assembly was driven by the hydrophobic effect. Cocrystals were obtained also at ≤1 M ammonium sulfate with ≤10 equiv of calixarene, but only at pH ≤ 4 where the protein is cationic. PhD student Kiefer Ramberg discovered a third cocrystal form in a low pH NMR sample after overnight incubation in the fridge. Each of the three crystal forms involved calixarene complexation of adjacent residues Val13 and Lys34 (Figure 6). The calixarene adopted conformations ranging from highly puckered to fully extended (pleated loop). The puckered conformation was molded neatly while the extended conformation was bound tangentially to the protein surface. Furthermore, the extended conformation occurred within a calixarene dimer (Figure 6b). Interestingly, the crystals obtained at high ammonium sulfate were densely packed, while the low pH crystals were porous (section 7).^4^ NMR studies revealed negligible calixarene binding at pH 6 but significant binding at pH 4. Two of the six acidic residues in RSL had elevated p*K*_a_ values in the presence of sulfonato-calix[8]arene suggesting pH-triggered assembly. The crystals provided supporting evidence, as the two acidic residues participated in calixarene complexation. Remarkably, one of the crystal forms accommodated different mutants, including an arginine-enriched and highly cationic variant (RSL-R_8_) that did not require low pH for cocrystallization.

With *C*_3_ symmetry, RSL presents three equivalent surfaces with interesting consequences for protein encapsulation by calix[8]arene. Each structure comprised the trimer masked to varying degrees with at least 6 calixarenes (Figure 6). Recently, large quaterphen[*n*]arenes, synthetically more challenging than calixarenes, have been used to encapsulate antimicrobial peptides.^76^

## 7 Protein–Calixarene Frameworks

**Figure 7:**
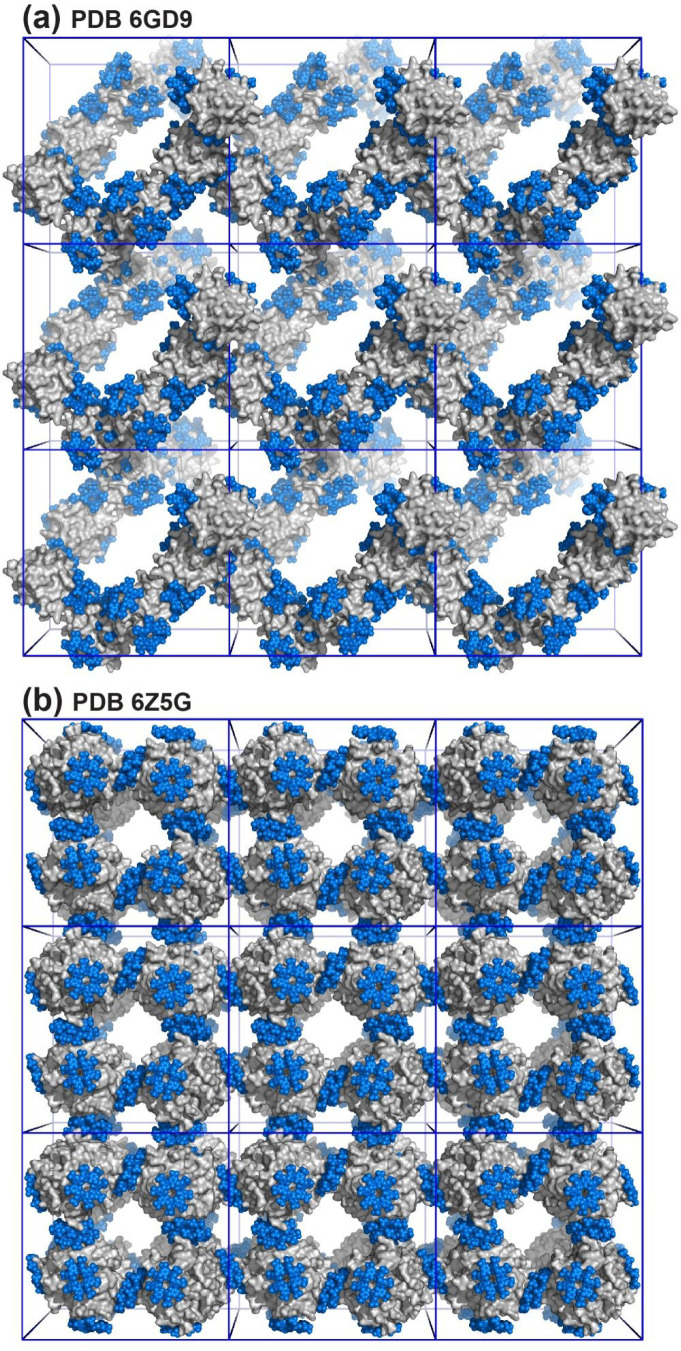
Porous frameworks of (a) cytochrome *c* (*P*4_3_2_1_2) and (b) RSL (*I*23) with unit cell axes *a* = *b* ≈ 10 nm. All interfaces are mediated by sulfonato-calix[8]arene.

With the developments from supramolecular to materials chemistry, the focus of molecular recognition has shifted from discrete complexes to frameworks, especially porous crystals. A similar shift is evident here. Although originally investigated as enzyme mimics and later as receptors for protein surfaces, it transpires that calixarenes are versatile scaffolds for constructing protein frameworks.^1,3,4^ Porous frameworks with solvent contents exceeding 50% were obtained by cocrystallization of cytochrome *c* with phosphonato-calix[6]arene,^61^ sulfonato-calix[6]arene,^62^ sulfonato-calix[8]arene,^1,3^ or the octa-anionic calix[4]arene.^55^ RSL and sulfonato-calix[8]arene also yielded highly porous structures.^4^ Here, I compare two such structures to illustrate framework fabrication by calixarenes (Figure 7).

In the presence of ≥3 equiv of sulfonato-calix[8]arene, cytochrome *c* forms an exceptionally porous framework with 85% solvent content.^1,3^ This diamondoid structure is held together exclusively by protein–calixarene–protein junctions and contains 3–10 nm wide cavities, suggesting the possibility of accommodating additional protein. The framework has no protein–protein contacts as each protein is substantially masked by the calixarene. Interestingly, one calixarene is highly solvent exposed, pointing into the crystal void. We wondered if this site could accommodate additional guests. Sylvain Engilberge tested ternary cocrystallization of protein, calixarene, and tetracationic spermine (structurally analogous to the dilysine motif), yielding two new structures with framework duplication and decreased porosity.^3^ RSL and sulfonato-calix[8]arene, at pH ≤ 4, also yielded two porous crystal forms devoid of protein–protein interfaces. A particularly aesthetic structure in the high symmetry (and rare), cubic space group *I*23 had 66% solvent content.^4^ Crystals in space group *P*3 were similarly porous and were obtained under trivial conditions in the absence of precipitant. This crystal form accommodated the cationic arginine-enriched RSL-R_8_, with only minor changes to the assembly despite an altered calixarene binding site. It remains to be seen what functionality can be achieved in these crystals, but efforts in other laboratories are promising.^77^

Porous protein assemblies provide a basis for new types of biocompatible and sustainable materials with broad applications. Different preparation strategies are in development,^77^ including designed protein assembly^78^ and the repurposing of natural protein cages.^79^ Macrocycle-mediated protein assembly confers advantages such as ease of fabrication and enhanced functionality via host–guest chemistry.^3,4^

## 8 Concluding Remarks

Almost 50 years ago, Cram and Cram wrote “the host molecule is the larger, and the guest molecule is the smaller of the two.”^80^ Host–guest chemistry of sulfonato-calix[*n*]arenes began with the trimethylanilinium cation^6−8^ and progressed to amino acids^13−18,30,31^ and later to proteins.^1−4,19,40^ Individual side chain encapsulation has led to protein encapsulation by multiple calixarenes, in which the “guest” is >10-fold larger than the “host.” What began as investigations of protein surface recognition (binary interactions) evolved to protein assembly (oligomerization) and crystallization (extended frameworks).^1,10,40^ Simple conditions containing PEG and a buffer/salt are sufficient to obtain cocrystals of cationic proteins with variously functionalized anionic calix[4]arenes.^2,40,45,59^ To date, success has been achieved with calixarenes bearing sulfonate or phosphonate substituents, but not carboxylates. Cationic calixarenes, although established protein binders,^23,24^ have yet to be cocrystallized with a protein. For example, guanidinio-containing calix[4]arenes have resisted cocrystallization, despite valiant efforts by PhD student Marta Giuliani. It remains to be seen what other functional groups may prove favorable. For example, the benzyl-sulfonate “extended arm” calix[8]arene formed a trimeric macrocycle stack leading to a new protein binding mode.^70^ And framework fabrication has taken a new direction with thiacalixarene, which enables dual macrocycle-/metal-mediated assembly and great scope for generating metal clusters in combination with proteins.^64^ The relatively simple and commercially available sulfonato-calix[8]arene has proven to be a versatile scaffold, mediating oligomerization, such as tetramerization of cytochrome *c*,^1^ enabling framework fabrication, such as the highly porous cubic assembly of RSL,^4^ or encapsulating individual proteins. Much remains to be discovered in protein assembly and encapsulation by macrocycles. Increased collaboration between biochemists and supramolecular chemists will lead to valuable advances, especially in the area of biomaterials.
